# WiFi-Based Driver’s Activity Monitoring with Efficient Computation of Radio-Image Features

**DOI:** 10.3390/s20051381

**Published:** 2020-03-03

**Authors:** Zain Ul Abiden Akhtar, Hongyu Wang

**Affiliations:** Faculty of Electronic Information and Electrical Engineering, Dalian University of Technology, Dalian 116023, China; zain_akhtar@outlook.com

**Keywords:** Channel State Information (CSI), wireless sensing, activity recognition, driver distraction, efficient computation, sparse auto-encoder

## Abstract

Driver distraction and fatigue are among the leading contributing factors in various fatal accidents. Driver activity monitoring can effectively reduce the number of roadway accidents. Besides the traditional methods that rely on camera or wearable devices, wireless technology for driver’s activity monitoring has emerged with remarkable attention. With substantial progress in WiFi-based device-free localization and activity recognition, radio-image features have achieved better recognition performance using the proficiency of image descriptors. The major drawback of image features is computational complexity, which increases exponentially, with the growth of irrelevant information in an image. It is still unresolved how to choose appropriate radio-image features to alleviate the expensive computational burden. This paper explores a computational efficient wireless technique that could recognize the attentive and inattentive status of a driver leveraging Channel State Information (CSI) of WiFi signals. In this novel research work, we demonstrate an efficient scheme to extract the representative features from the discriminant components of radio-images to reduce the computational cost with significant improvement in recognition accuracy. Specifically, we addressed the problem of the computational burden by efficacious use of Gabor filters with gray level statistical features. The presented low-cost solution requires neither sophisticated camera support to capture images nor any special hardware to carry with the user. This novel framework is evaluated in terms of activity recognition accuracy. To ensure the reliability of the suggested scheme, we analyzed the results by adopting different evaluation metrics. Experimental results show that the presented prototype outperforms the traditional methods with an average recognition accuracy of 93.1% in promising application scenarios. This ubiquitous model leads to improve the system performance significantly for the diverse scale of applications. In the realm of intelligent vehicles and assisted driving systems, the proposed wireless solution can effectively characterize the driving maneuvers, primary tasks, driver distraction, and fatigue by exploiting radio-image descriptors.

## 1. Introduction

Driver’s safety is arguably a captivating task in fast-paced intelligent vehicles. During the previous decades, a large number of traffic accidents are reported due to driver distraction or fatigue [1]. Distraction reduce the driver’s perception and decision making capability by diverting his/her attention from the primary task of driving to secondary activities [2]. In-vehicle entertainment systems (audio/video players) and gadgets (GPS and mobile communication) are among the leading causal factors of driver distraction [3,4]. Moreover, a driver may be distracted because of engagement in eating/drinking, smoking or talking to the passengers during driving [5,6]. The challenging problem is how to measure the driver’s concentration on the essential task of driving.

Massive attempts have already been done for driver monitoring using camera [7,8] or sensors [9,10]. However, these traditional methods have several key limitations in practical scenarios [11]. For example, camera-based methods cannot work properly in darkness and through the walls [12]. Whereas, sensor-based methods are intrusive to the human body and need burdensome installation [12]. The radar-based system is another possible solution for driver monitoring using the reflections of radio waves [13]. However, conventional radar systems are usually used outside the vehicles, while short-range fine-grained radar systems have not been so far practically implemented [14]. In contrast, WiFi-based activity recognition is a promising solution to overcome all these limitations. The WiFi-based device-free wireless systems are non-intrusive to users, easy to install [15,16], and work properly in both line-of-sight and non-line-of-sight scenarios.

During the previous few years, WiFi-based device-free localization, activity, and gesture recognition systems have been successfully used in various applications including assisted living, health monitoring, and emergency surveillance [15,16,17,18,19,20,21,22]. WiFi-based wireless scheme opened a new window for scientists to further investigate device-free activity recognition for the safety benefits of drivers. In this context, several WiFi-based driver monitoring systems have been investigated with good recognition performance [14,23,24,25,26,27]. Despite all its prospects, the WiFi-based complete description of driver’s attention and inattention monitoring has not been deeply investigated thus far and is still a competitive task to solve. This paper explores the potential and capability of Channel State Information (CSI) of the WiFi signal for driver’s activity monitoring with efficient computation of radio-images. To achieve this goal, we have to face several challenges because the uncontrolled in-vehicle environment is quite different from indoor environments. We performed extensive experiments and designed the prototype to combat these challenges.

The emerging radio-image features for device-free localization and activity recognition rely on CSI data of WiFi signals influenced by the human body [28,29,30,31]. Although radio-image algorithms have achieved better recognition performance exploiting image feature descriptors, i.e., Gabor filter bank, but there are still many open problems. The major drawback of Gabor wavelet features is the expensive computational burden, which increases drastically, with the growth of irrelevant image information. Regarding dimensionality, Gabor wavelet features require large memory space with high computational complexity. This inspired us to discover an innovative solution for a computationally efficient method by selecting the discriminant components of WiFi CSI measurements for radio-image processing. The discriminant component is a general term used throughout the work as the most important WiFi CSI data values that participate well in the discrimination process.

In the proposed mechanism, we extract image features only from the discriminant components of radio-images. We are using both Gabor wavelet features and gray level statistical features. We particularly adopt the Gray Level Co-occurrence Matrix (GLCM), a widespread texture-based feature extraction method that we call gray level statistical features. Both feature extraction methods are successful and competent for texture-based recognition [32,33,34,35,36]. Afterward, for assessing the relevance of extracted features, we apply Auto-Encoder (AE) method. In the proposed scheme, Stacked Sparse Auto-Encoder (S-SAE) model is used to classify different attentive and inattentive activities. S-SAE is an emerging algorithm that is based on Auto-Encoder (AE) of artificial neural networks and has been thoroughly studied in literature with multiple variants [37,38,39,40]. In this novel research work, we implemented S-SAE in a semi-supervised manner for a complete description of the driver’s activities exploiting radio-image features.

In this paper, we focus on characterizing fifteen common driving activities of which six are treated as attentive activities comprising of normal driving tasks (4-driving maneuvers, 2-primary activities), while nine are regarded as inattentive activities (5-distraction and 4-fatigue activities), as described in Table 1. The attentive activities are divided into two groups, i.e., driving maneuvers (turning right, turning left, driving straight, steering corrections) and primary tasks (operating gear stick, mirrors checking). Inattentive activities are divided into two groups, i.e., distraction (eating, talking with a passenger, talking or listening on the mobile phone, dialing a mobile phone, operating infotainment system) and fatigue (repeated yawning, head itching, face scratching, head nodding). The proposed mechanism leverages wireless channel variations that are readily available on commodity WiFi devices in the form of CSI measurements [41]. Intuitively, the WiFi CSI channel variations are caused by driver’s activities in a WiFi coverage area. As per our knowledge, this is the first approach towards device-free driver’s activity monitoring using WiFi-based radio-image features. This efficient scheme can improve the activity recognition performance significantly with a less computational burden. The main contributing aspects of this research work are as follows:

- We proposed a computational efficient WiFi-based driver’s activity monitoring system exploiting the discriminant components for radio-image processing.
- To validate the scalability of results, we conduct extensive experiments in promising application scenarios and comparative evaluation is performed with state-of-the-art methods.

The remainder of the paper is arranged as follows: In Section 2, we review the traditional techniques of device-free wireless activity recognition relevant to the presented work. Section 3 highlights the overview of the suggested method and system architecture with the basic concepts of CSI. Section 4 is concerned with the complete flow of the system methodology. Section 5 demonstrates the experimentation settings and performance evaluation. Section 6 validates the results in discussion with some limitations regarding the proposed solution. Finally, we conclude the presented work with some future suggestions in Section 7.

## 2. Related Work

In this section, we will review the traditional techniques relevant to the presented work with three different aspects, i.e., WiFi-based efficient activity recognition, WiFi-based radio-image processing, and WiFi-based driver’s activity/gesture recognition.

In general, Wi-Alarm [42] investigated a low computational cost WiFi-based intrusion detection system by eliminating data pre-processing cost. A WiFi-based elderly activity recognition system was demonstrated in Reference [12]. To reduce data dimensions and alleviate the computational complexity, they choose Principal Component Analysis (PCA) for useful information across all WiFi CSI streams and represented CSI vector as the mean of thirty subcarriers. This mechanism is not suitable for radio imaging, because it may scale down the importance of most relevant components. WiFall [43] detected WiFi-based abnormal behavior depending on the local outlier factor. The authors of Reference [44] examined a CSI-based human Activity Recognition and Monitoring system (CARM) using the CSI speed model and CSI activity model. They described a correlation between a specific activity and human body movement speed. During the previous decades, WiFi-based micro-activity recognition [45] and intrusion detection systems [18,46] have been explored with very good performance. Wireless indoor localization has been permeated into an advanced era of life [47]. Recently, a WiFi-based training-free localization system has been emerged with very good results [48].

During the previous few years, WiVi [49] introduced through-wall motion-imaging based on multi-antenna techniques. However, this system requires a specialized receiver to deal with Orthogonal Frequency Division Multiplexing (OFDM) technique. TW-See [50] demonstrated opposite-robust PCA (Or-PCA) technique for passive human activity recognition through the wall with commodity WiFi devices. In recent years, WiFi-based radio-image processing [28] has achieved valuable activity recognition performance using Gabor filters. The authors of Reference [29] investigated a vision-based method to classify human activities exploiting radio-images of WiFi CSI. Further improvement in WiFi vision-based method has been achieved using singular value decomposition for location dependency removal [30]. Human action recognition along with user identification is a break-through in WiFi vision-based methods [31]. These schemes represented CSI-transformed images as Gabor coefficients and used statistical methods to reduce the computational complexity of Gabor wavelet features. Although we are also using statistical methods but different from others, we are extracting low-cost image features only from the discriminant components.

Thus far, limited work is reported in the literature on WiFi-based driver’s activity or gesture recognition. In this context, WiDriver [24] is based on the driver’s hand movements to characterize driving actions. WiFind [23] is suitable only for driver fatigue detection. SafeDrive-Fi [25] investigated dangerous driving action recognition using CSI of WiFi signals. The authors of Reference [27] examined a novel WiFi-based wireless driver head tracking system. To track the driver’s head orientations, they exploit CSI of the phone’s WiFi signal and implemented a position-orientation joint profiling technique to quickly build CSI profile. WiDrive [14] demonstrated a real-time driver’s activity recognition system based on CSI variations of WiFi signals. This system is suitable to recognize small-scale takeover-related in-car activities with good recognition results. Recently, WiFi-based gesture recognition for vehicular infotainment system has been investigated with good recognition results [26]. WiFi-based vehicular technology has been approached to the ability of vehicle speed estimation [51]. Different from existing works, the presented device-free WiFi-based innovative framework is suitable for both driver’s attention and inattention monitoring including primary tasks, driving maneuvers, driver distraction, and fatigue recognition.

## 3. System Overview

In this section, we will highlight the presented prototype and overview of system flow with some relevant important features of WiFi CSI.

### 3.1. Efficient Computation of Radio-Image Features

CSI channel matrix of the WiFi signal has a close resemblance to the image matrix [28]. However, we cannot directly convert the WiFi CSI channel matrix into the image matrix, because WiFi CSI raw matrix contains a lot of irrelevant information. The key challenge is how to detect the sensitive WiFi CSI segment of transformed radio-images that contains information about the driver’s activities and extract the discriminative features to differentiate these activities. To achieve this goal, we adopt cumulative moving variance to detect the presence of activity in the WiFi CSI channel matrix [52]. Firstly, we propose and formulate a model for activity profile extraction and then we select discriminant components of activity profile. Afterward, we transform the optimized CSI matrix into the radio-image matrix that contains only the discriminant components relevant to the activity. Finally, we extract image features from these discriminant components of radio-images.

The proposed sophisticated solution is based on a simple fact that all components of radio-image do not participate well in the discrimination process. Meanwhile, we cannot ignore the importance of any representative component, as each component carries a different level of discriminative information. In this proposed scheme, the discriminant components are carefully selected based on unique variations in WiFi CSI measurements caused by driver activities. By selecting discriminant components, we are decreasing the computational complexity of computed coefficients. In general, the total number of discriminant components is much less as compared to the actual information of the activity profile. The Gabor and GLCM features are extracted from CSI-transformed radio-images. The extracted coefficients are further analyzed using Stacked Sparse Auto-Encoder (S-SAE) to get more optimized features. Thereafter, optimum features are used to train a neural network classifier and eventually activities are classified. The detailed description of each step is given in Section 4.

### 3.2. CSI Overview

In this section, we demonstrate the preliminaries about CSI of the WiFi signal. The proposed system analyzes the impact of driver’s activities on a WiFi channel in the form of CSI measurements. Existing WiFi devices support widely used Multiple-Input Multiple-Output (MIMO) and Orthogonal Frequency Division Multiplexing (OFDM) mechanism. These systems consist of multiple transmitting (Tx) and receiving (Rx) antennas, exploiting IEEE 802.11n protocol. Fine-grained CSI data of WiFi signal reveals the information about how signals propagate from transmitter to receiver. Each Tx-Rx antenna pair of WiFi CSI system supports 30 OFDM subcarriers to record channel variations, available on commercial WiFi devices in the form of CSI measurements [41].

Throughout our experiments, the transmitter is an Access Point (AP) with 802.11n enabled protocol. On the other hand, the receiver is a laptop equipped with Intel 5300 NIC to collect physical layer CSI data of the WiFi signal. An ordinary narrowband flat-fading channel for packet *i*, exploiting OFDM and MIMO technology can be represented as:

(1) $$Y_{i}=H_{i}X_{i}+N_{i},\;i\in \left[1,N\right]$$

where *N* denotes the total number of received packets, $N_{i}$ indicates the Gaussian noise vector, $H_{i}$ stands for CSI channel matrix, $X_{i}$ and $Y_{i}$ refer to transmitted and received signals respectively.

Let $N_{Tx}$ and $N_{Rx}$ are designated for the number of transmitting and receiving antennas respectively. Each stream in CSI channel matrix of WiFi signal consists of $N_{Tx}\times N_{Rx}\times 30$ complex values. For each Tx-Rx antenna pair, WiFi CSI channel matrix *H* can be described as:

(2) $$H_{i}=\left[h_{1},h_{2},\ldots ,h_{30}\right]\;i\in \left[1,N\right]$$

where *h* represents a complex value that depicts the information related to the phase and amplitude. Mathematically, it can be estimated as:

(3) $$h=\left|h\right|e^{jsin\{∠h\}}$$

where ∠h represents the phase information, and |h| indicates the amplitude.

### 3.3. System Architecture

**Figure 1:**
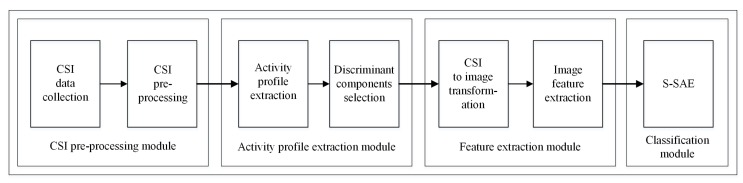
System architecture.

In this section, we will overview the system architecture of the suggested scheme. The proposed model is shown in Figure 1 that consists of four basic modules including (1) CSI pre-processing module, (2) activity profile extraction module, (3) feature extraction module, and (4) classification module.

CSI pre-processing module collects CSI data of the WiFi signal and processes this data using basic filtering techniques. The activity profile extraction module detects the presence of activity and extracts discriminant components related to that activity. Feature extraction module is responsible to transform CSI discriminant values into radio-images and extract image features including Gabor and gray level statistical features. In the classification module, recognition is performed using an efficient classification method. The detailed description of each module is given in Section 4.

## 4. Methodology

In this section, we will explain the complete flow of the system methodology.

### 4.1. CSI Pre-Processing Module

The proposed prototype leverages WiFi ambient signal as the information source to analyze the influence of driver activities on a wireless channel. The channel variations are readily available in the form of CSI measurements on commercial WiFi devices [41]. Particularly, in this section, we will use the term CSI as raw-CSI of the WiFi signal alternatively. CSI received signal carries useful information embedded with undesirable noises. Firstly, we remove the DC component from each CSI stream by subtracting the constant offset [44]. The corresponding constant offset is estimated by a long-term averaging over CSI stream [16]. The human activities have a relatively low frequency as compared to the noise [18]. Therefore, we filter out high frequencies from the received signal. To remove high-frequency noise, we apply second order low pass Butterworth filter at subcarrier level. We adjust packets sampling rate ($F_{s}$) at 80 packets/second, same as normalized cutoff frequency $w_{n}=2\pi f/F_{s}=0.025\pi \;rad/s$.

CSI raw phase measurements perform randomly because of hardware differences and unsynchronized time clock between transmitter and receiver [53]. To eliminate phase offset and extract the actual phase, we perform phase calibration.

#### 4.1.1. Phase Calibration

Let $∠h_{i}$ and $∠\hat{h_{i}}$ are true phase and measured phase respectively for *i*th subcarrier. The true and measured phase are related as:

(4) $$∠\hat{h_{i}}=∠h_{i}+2\pi \frac{n_{i}}{N}\Delta t+\beta +z$$

where $n_{i}$ represents the subcarrier index of $i^{th}$ subcarrier, *N* denotes the size of FFT, Δt is the time lag, *z* indicates the random noise and β stands for unknown phase offset. It is difficult to estimate the exact value of Δt and β for each packet. However, by applying a simple transformation, we can calculate the same value of Δt and β each time. Phase error ($2\pi \frac{n_{i}}{N}\Delta t+\beta$) may be considered to be a linear function of subcarrier index $n_{i}$. We can formulate two parameters *a* and *b* for the calibration of phase error as:

(5) $$a=\frac{∠{\hat{h}}_{k}-∠{\hat{h}}_{1}}{n_{k}-n_{1}}$$

(6) $$b=\frac{1}{k}\sum_{j=1}^{k}∠{\hat{h}}_{j}$$

where *a* represents the slope of phase, while *b* stands for the offset across the entire frequency band. We subtract a linear term $an_{i}+b$ from raw phase $∠{\hat{h}}_{i}-an_{i}$ to get the sanitized phase $∠{\tilde{h}}_{i}$ as:

(7) $$∠{\tilde{h}}_{i}=∠{\hat{h}}_{i}-an_{i}-b$$

The phase sanitization is performed on all the subcarriers and re-assembled according to the corresponding amplitudes.

#### 4.1.2. Amplitude Information Processing

Afterward, we propose to apply a Weighted Moving Average (WMA) algorithm over CSI streams to eliminate the outliers by following the procedure as described in [43].

Let $h_{t}$ denotes CSI value at specific time interval *t*, then the expression for WMA can be written as:

(8) $$h_{t}^{\prime }=\frac{\left[m\times h_{m}+\left(m-1\right)\times h_{\left(m-1\right)}+\ldots +1\times h_{1}\right]}{m+(m-1)+\ldots +1}$$

where $h_{t}^{\prime }$ is the averaged CSI value at time *t*. New value $h_{t}^{\prime }$ has the weight value of *m* that decides to what degree the current value relates to historical data.

### 4.2. Activity Profile Extraction Module

This module takes the pre-processed WiFi CSI measurements and detects the presence of activity to select discriminant components related to the activity.

#### 4.2.1. Activity Profile Extraction

The activity profile extraction is based on cumulative moving variance, as described in Reference [52]. Suppose *H* is the WiFi CSI matrix after noise removal and sanitization. We organize CSI values in a matrix with dimensions T×R, where *T* is the number of CSI streams received and *R* is the window size. The window size R corresponds to the sampling rate and duration of each activity. We divide the matrix *H* in a row-order into small bins. Let $T_{b}$ is the total number of bins, where each bin comprises of *n* number of CSI measurements, such that n∈H. Assume a new matrix $N_{m}$ with dimensions equal to T×n is reconstructed from each bin *m* and $N_{m}\left(1\leq m\leq T_{b}\right)$. We measure the variance $\sigma_{m}$ for each bin and the cumulative moving variance (V) can be estimated as:

(9) $$V=\sum_{m=1}^{T_{b}}\sigma_{m}$$

The next step is to investigate the cumulative moving variance to analyze whether it contains activity-related information or not. For this purpose, we set a threshold $\tau_{th}$ and compare the maximum value of V with $\tau_{th}$ for activity related component selection. Mathematically,

(10) $$\Psi =\left\{\begin{array}{cc} 1, & max\left(V\right)>\;\tau_{th}\\ 0, & otherwise \end{array}\right.$$

where Ψ represents the presence of activity information. If Ψ is set to 1, it means activity is detected and we apply discriminant component selection, otherwise, Ψ is set to zero. The value of threshold $\tau_{th}$ is empirically selected based on the variance of preliminary measurements that varies with the activities.

#### 4.2.2. Discriminant Components Selection

After realizing the activity profile, we examine the discriminant components involved in an activity by analyzing the second Eigen-vector and second principal component [44]. First of all, we estimate the correlation matrix $N_{m}^{T}\times N_{m}$ for each bin *m* and Eigen-decomposition is applied to obtain Eigen-vectors. The discriminant component selection is performed by observing the variation in second Eigen-vector $q_{2}$ and the corresponding second principal component $h_{2}$. We need to calculate the mean of the first difference of $q_{2}$ and the variance of $h_{2}$. Mean of the first difference of $q_{2}$ is represented as ${\mu_{q}}_{2}$ and mathematically calculated as:

(11) $${\mu_{q}}_{2}=\frac{1}{T-1}\sum_{i=2}^{T}\left|q_{2}\left(i\right)-q_{2}\left(i-1\right)\right|$$

**Figure 2:**
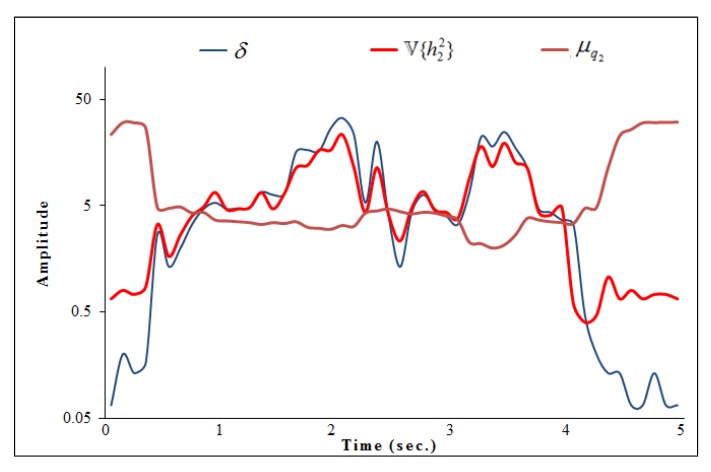
Discriminant components selection.

From the Figure 2, it is clear that as the activity is performed, $q_{2}$ varies smoothly resulting in a smaller value of ${\mu_{q}}_{2}$. Whereas, in the presence of activity, the variance of $h_{2}$ represented as $V\{h_{2}^{2}\}$ has a larger value. Therefore, we focus on the ratio (δ) between ${\mu_{q}}_{2}$ and $V\{h_{2}^{2}\}$ to decide the discriminant components of activity. To automatically recognize the discriminant components of an activity, we set a threshold $\eta_{th}$ dynamically using an exponential moving average algorithm [54]. We compare the ratio δ with threshold $\eta_{th}$ to obtain discriminant component value ϕ as:

(12) $$\phi =\left\{\begin{array}{cc} 1, & \mathrm{if}\;\delta \geq \;\eta_{th}\\ 0, & \mathrm{otherwise} \end{array}\right.$$

The value of ϕ decides whether it is a discriminant component or not, by following conditions:

(13) $$\mathrm{if}\;\;\phi =\left\{\begin{array}{cc} 1, & \mathrm{discriminant}\;\mathrm{component}\\ 0, & \mathrm{not}\;a\;\mathrm{discriminant}\;\mathrm{component} \end{array}\right.$$

The non-discriminant components are discarded from the WiFi CSI channel matrix, and we obtain optimized CSI values with discriminant component of the activity profiles.

### 4.3. Feature Extraction Module

The feature extraction module transforms the optimized CSI values into radio-images and performs efficient discriminant image feature extraction. We particularly select two classical types of features, i.e., Gabor wavelet features and gray level statistical features (GLCM). We are using both amplitude and phase measurements of WiFi CSI data.

#### 4.3.1. CSI to Image Transformation

We transform the optimized CSI data into radio-images with discriminant components only that carries sufficient discriminative information. We construct two-dimensional (2D) radio-images by taking time (magnitude strength) on the x-axis and channel on the y-axis, as described in Reference [28]. Let R be the obtained CSI transformed radio-image. We apply the Gabor filter and GLCM feature extraction methods to achieve informative features. Since the number of discriminant components is very less as compared to actual information. As a result, we will obtain efficient computation of image feature descriptors.

#### 4.3.2. Gabor Feature Extraction

CSI-transformed image matrix has a great resemblance to textual appearances. Gabor filter is a successful texture or image descriptor in face recognition and image processing [32,33,34]. Two dimensional Gabor filter as complex sinusoidal modulated Gaussian function can be explained as:

(14) $$G\left(x,y,\lambda ,\theta_{k},\sigma_{x},\sigma_{y}\right)=\frac{1}{2\pi \sigma_{x}\sigma_{y}}exp\left\{-\frac{1}{2}\left[\frac{x_{\theta_{k}}^{2}}{\sigma_{x}^{2}}+\frac{y_{\theta_{k}}^{2}}{\sigma_{y}^{2}}\right]\right\}\times exp\left[k.\frac{2\pi x_{\theta_{k}}}{\lambda }\right]$$

such that

(15) $$x_{\theta_{k}}=xcos\theta_{k}+ysin\theta_{k}$$

(16) $$y_{\theta_{k}}=ycos\theta_{k}-xsin\theta_{k}$$

where $\theta_{k}$ denotes the orientation and λ stands for the wavelength. $\sigma_{x}$ and $\sigma_{y}$ represent the standard deviation of the Gaussian envelop along the x-axis and y-axis respectively.

To enable the filter to extract frequency information that is dependent on orientation, we set different values of λ and $\theta_{k}$. For any given value of λ and $\theta_{k}$, Gabor features are extracted by the convolution of a filter with radio-image. This mechanism can be described as:

(17) $$\hat{G}\left(x,y\right)=R\left(x,y\right)\times G\left(x,y\right)$$

After convolution process, we obtain Gabor features $\hat{G}\left(x,y\right)$ that is equal to the size of radio-image R(x,y). The performance of Gabor coefficients $\hat{G}\left(x,y\right)$ cannot be effectively used due to high computational cost. We adopt two statistics, i.e., mean (μ) and variance (σ) to retain the complementary features of Gabor coefficients and reduce the computational complexity of Gabor coefficients, represented as:

(18) $$\mu =\frac{1}{LW}\sum_{x=1}^{L}\sum_{y=1}^{W}\hat{G}\left(x,y\right)$$

(19) $$\sigma =\frac{1}{LW}\sum_{x=1}^{L}\sum_{y=1}^{W}{\left(\hat{G}\left(x,y\right)-\mu \right)}^{2}$$

where *L* and *W* denote the length and width of radio-image respectively. In total, we have $1\times 2\lambda_{m}\theta_{n}$ Gabor feature vector G that corresponds to *m* number of wavelengths and *n* number of orientations, described as:

(20) $$G=[\left(\mu_{1},\sigma_{1}\right),\ldots ,\left(\mu_{\lambda_{m}},\sigma_{\theta_{n}}\right)]$$

In this experiment, the dimension of Gabor-filtered vector is based on 40 Gabor kernels; with best suited 5-spatial wavelengths ($2\sqrt{2},4,4\sqrt{2},8,8\sqrt{2}$) and 8-orientations (from 0 to $\frac{7\pi }{8}$ in uniform steps of $\frac{\pi }{8}$) using 19×19 Gabor filters.

#### 4.3.3. Statistical Feature Extraction

We adopt the Gray Level Co-occurrence Matrix (GLCM) method to compute texture-based statistical features. The GLCM extracts statistical meaningful features by constituting a matrix with the distances and orientations between pixels [35]. In this simple approach, concurrence time of the same gray level pixel pairs are examined to calculate the relationship between the reference pixel and the neighboring pixels in an image.

Assuming *i* and *j* are gray level values of pixels pair in an image separated by a distance *d*. The GLCM matrix consists of the probability values represented by P(i,j|d,θ) for pixels pair at distance *d* and orientation θ. Therefore, P(i,j|d,θ) demonstrates the concurrence time for pixels pair. Every element of the GLCM matrix is characterizing the occurrence probability of gray level value for each pixel. Let *N* is the sum of all element values describing the total number of concurrent time of gray level values, then normalized GLCM is described as:

(21) $$P_{n}\left(i,j|d,\theta \right)=\frac{P(i,j|d,\theta )}{N},\;where\;\theta =\left\{0,\frac{\pi }{4},\frac{\pi }{2},\frac{3\pi }{4}\right\}$$

In this paper, we particularly select four co-occurrence statistics including entropy, inverse difference moment, energy and correlation, as described in Reference [36].

1. Entropy: It measures the randomness and disorder of the image. Non-uniform texture corresponds to a high value of entropy. Mathematically, entropy (R) is defined as:
  (22) $$R=-\sum_{i}\sum_{j}P_{n}\left(i,j|d,\theta \right)log\left[P_{n}\left(i,j|d,\theta \right)\right]$$
2. Inverse difference moment: It is defined as the measure of the smoothness of an image. Inverse difference moment (I) is mathematically represented as:
  (23) $$I=\sum_{i}\sum_{j}\frac{P_{n}\left(i,j|d,\theta \right)}{1+{\left(i-j\right)}^{2}}$$
3. Energy: It measures the occurrence of pixel pairs that are repeated in an image. Mathematically, energy (E) is defined as:
  (24) $$E=\sum_{i}\sum_{j}{\left[P_{n}\left(i,j|d,\theta \right)\right]}^{2}$$
4. Correlation: It is the similarity measure between a pixel of a specific region and its neighboring pixel in an image. Mathematically, correlation (C) can be represented as:
  (25) $$C=\frac{\sum_{i}\sum_{j}\left(i\times j\right)P_{n}\left(i,j|d,\theta \right)-\mu_{k_{1}}\mu_{k_{2}}}{\sigma_{k_{1}}\sigma_{k_{2}}}$$

where

(26) $$\mu_{k_{1}}=\sum_{i}\sum_{j}i\times P_{n}\left(i,j|d,\theta \right),\;\mu_{k_{2}}=\sum_{i}\sum_{j}j\times P_{n}\left(i,j|d,\theta \right),$$

(27) $$\sigma_{k_{1}}=\sum_{i}\sum_{j}{\left(i-\mu_{k_{1}}\right)}^{2}\times P_{n}\left(i,j|d,\theta \right),\;\sigma_{k_{2}}=\sum_{i}\sum_{j}{\left(i-\mu_{k_{2}}\right)}^{2}\times P_{n}\left(i,j|d,\theta \right),$$

By following the obtained metrics, the GLCM features for distance *d* and orientation θ can be explained as:

(28) $$M_{d,\theta }=\left[R_{d,\theta },I_{d,\theta },E_{d,\theta },C_{d,\theta }\right]$$

In total, we have $1\times 4d_{m}\theta_{n}$ GLCM feature vector M corresponding to *m* number of distances and *n* number of orientations, defined as:

(29) $$M=[M_{1,1},\ldots ,M_{d_{m},\theta_{n}}]$$

Finally, we carefully concatenate Gabor and GLCM features to get comprehensive features for the classifier.

### 4.4. Classification Module

In this section, we will discuss the proposed semi-supervised deep learning model that is based on Stacked Sparse Auto-Encoder (S-SAE). In conventional algorithms, unlabeled data cannot be used for classification purposes. The presented scheme is using both labeled and unlabeled data to enhance the recognition accuracy of the system. We adopt Stacked Sparse Auto-encoder (S-SAE) that is linked with Neural Network (NN) classifier to build a deep learning model. Classification is based on a semi-supervised method with predicted labels of unlabeled data, as demonstrated in Reference [40].

**Figure 3:**
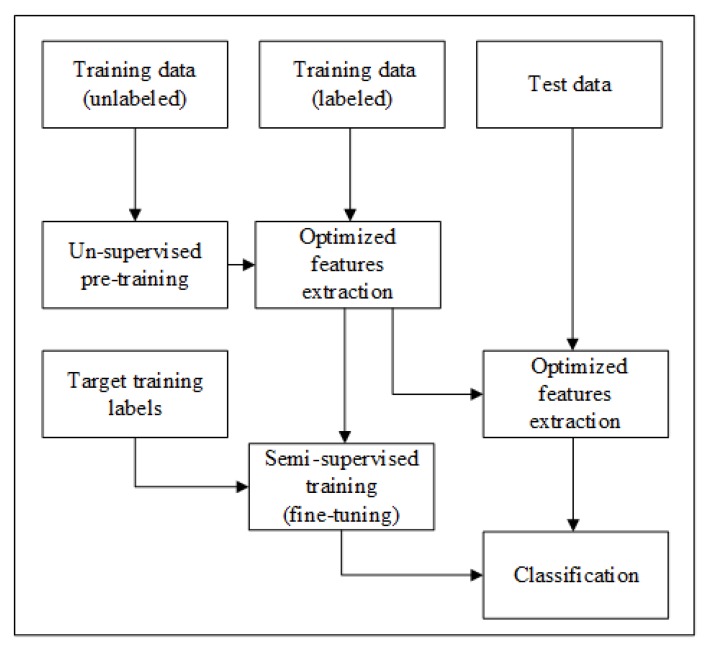
Complete flow of semi-supervised framework.

This semi-supervised classification mechanism is based on the unsupervised feature extraction method and the supervised classification method. In this technique, we are taking the advantages of both labeled and unlabeled data for better prediction performance. The complete flow of suggested semi-supervised learning framework is illustrated in Figure 3. Firstly, unlabeled training data is used for unsupervised feature learning at the pre-training stage. In the next stage, optimized features are extracted using labeled data and unsupervised pre-training features that are further used in semi-supervised training of the classifier. Afterward, fine-tuning is performed to enhance the performance of S-SAE. Finally, fine-tuned features are used to train the classifier and test features are used to check the recognition accuracy. By following this technique, we are using the ability of S-SAE to deal with unlabeled information in the feature extraction scheme, and consequently, more effective results are obtained in the subsequent classification stage.

#### Stacked Sparse Auto-Encoder (S-SAE)

The auto-encoder is an unsupervised technique for the neural network model that reconstructs the high dimensional input data and converts it into low dimensional outputs representation [55]. A Stacked Sparse Auto-Encoder (S-SAE) consists of a hierarchy of multiple layers of basic Sparse Auto-Encoder (SAE). SAE is based on the idea of sparsity, where AE learns a representation and simultaneously impose sparsity on the activation of hidden units. The number of hidden units is set to be less than the number of input units for data dimension reduction. In S-SAE the output of each layer is connected to the input of successive layer, where each layer is learned by a single SAE.

The presented stacking mechanism works on a simple principle, where unlabeled data is used to initialize the weights blocks using unsupervised learning in a greedy-layer manner. In a greedy-layer technique, the first-level parameters are obtained from the first AE using original input data. These representations are then used as first-level features that are used to train second AE and similarly next level parameters are obtained. This mechanism continues until the last AE is trained and sufficient features are extracted with low feature dimensions. In this way, feature extraction is done based on the stacking of AEs for pre-training. Afterward, the network is fine-tuned using back-propagation where labeled data is used.

**Figure 4:**
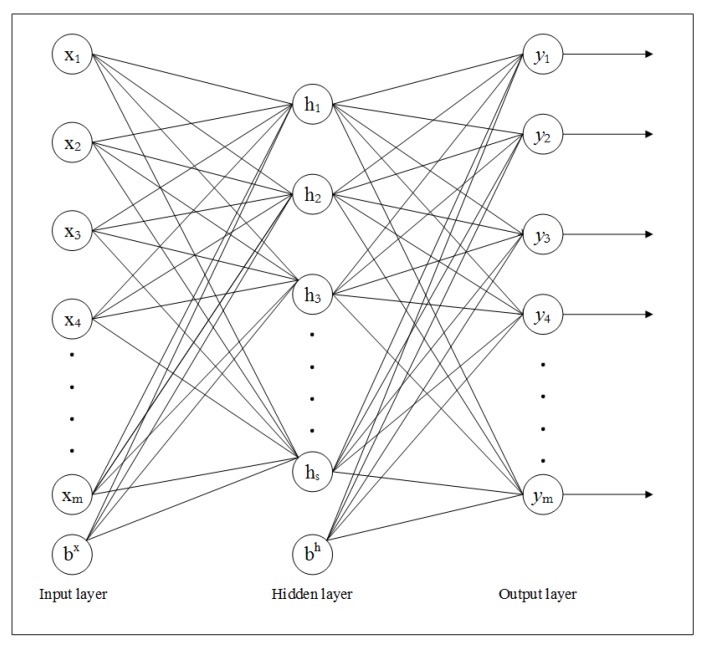
Basic structure of SAE.

**Figure 5:**
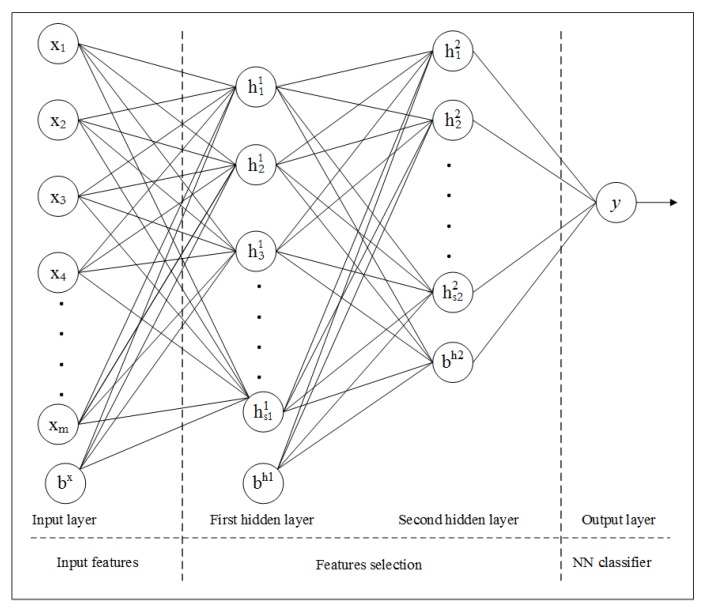
Structure of presented S-SAE model.

Let $\left\{x_{1},x_{2},\ldots ,x_{m}\right\}$ is the set of inputs, $\left\{h_{1},h_{2},\ldots ,h_{s}\right\}$ is the set of hidden layer’s outputs, and $\left\{y_{1},y_{2},\ldots ,y_{m}\right\}$ is the set of corresponding outputs of the output layer. If $b^{x}$ and $b^{h}$ denote the bias units to hidden and output layers respectively, then the basic structure of SAE is shown in Figure 4. In this implementation, we are using two-layered SAE that consist of two hidden layers as shown in Figure 5. The SAE attempts to learn the hypothesis function with unlabeled data as:

(30) $$h_{W,b}\left(x\right)=x$$

where *W* represents the weight matrix and *b* is the bias vector. Let we have $\left\{x^{1},x^{2},\ldots ,x^{n}\right\}$ training samples. The cost function of SAE is determined as:

(31) $$j\left(W,b\right)=\frac{1}{n}\sum_{i=1}^{n}\frac{1}{2}{∥h_{W,b}\left(x^{i}\right)-y^{i}∥}^{2}+\frac{\lambda }{2}\sum_{i=1}^{s_{l}}\sum_{j=1}^{s_{l+1}}{\left(W_{ji}^{\left(l\right)}\right)}^{2}+\beta \sum_{j=1}^{s_{l}}KL\left(\rho ∥{\hat{\rho }}_{j}\right)$$

where *n* is the total number of training samples, $x^{i}$ denotes the $i^{th}$ training sample of SAE input, and $y^{i}$ is the corresponding output. λ is the weight decay parameter, $s_{l}$ denotes the number of units in layer *l*, ${\left(W_{ji}^{\left(l\right)}\right)}^{2}$ represents an element in $W^{l}$. The term $\beta \sum_{j=1}^{s_{l}}KL\left(\rho ∥{\hat{\rho }}_{j}\right)$ stands for the sparsity penalty and $KL(\rho ∥{\hat{\rho }}_{j})$ is the Kullback-Leibler (KL) divergence function, where ρ represents the sparsity parameter and ${\hat{\rho }}_{j}$ denotes the average activation of hidden unit *j*. To maintain the sparsity of S-SAE, the Kullback-Leibler parameter keeps the activation of each neuron to be close to ρ. Suppose, we have $\left(x^{1},y^{1}\right),\left(x^{2},y^{2}\right),\ldots ,\left(x^{n},y^{n}\right)$, as training data set, then the cost function can be formulated as:

(32) $$j\left(W,b\right)=\frac{1}{2n}\sum_{i=1}^{n}{∥y_{W,b}\left(x^{i}\right)-y^{i}∥}^{2}$$

where $y^{i}$ and $y_{W,b}\left(x^{i}\right)$ represent the targeted and predicted values respectively. To complete the classification task, we have to reduce the cost function J(W,b), because activation of the hidden unit is dependent on *W* and *b*. The parameter (W,b) can be optimized by implementing the back-propagation algorithm on S-SAE-based deep learning classification model. Afterward, the parameters are fine-tuned to minimize the recognition error and the classification is finalized.

## 5. Experimentation and Evaluation

In this section, we will describe the experimental settings and performance evaluation of the proposed scheme.

### 5.1. Experimentation Settings

**Figure 6:**
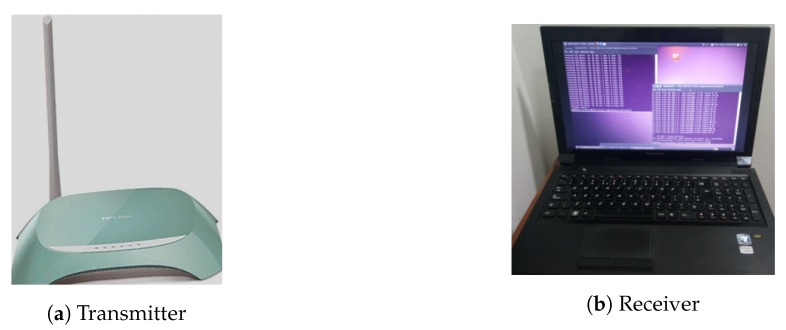
Experimentation equipment.

We conduct extensive experiments using off-the-shelf WiFi devices with 802.11n enabled protocols. In our experiments, a Lenovo laptop is used as a receiver with Ubuntu 11.04 LTS operating system. The receiver is equipped with a network interface card (NIC) Intel 5300 to collect WiFi CSI data with three antennas. The receiver connects to WiFi Access Point (AP); a TP-Link (TL-WR742N) router as transmitter operating at 2.4 GHz with a single antenna. The experimentation equipment is shown in Figure 6. The receiver can ping the AP at a rate of 80 packets/s. The system is generating 3 CSI streams of 30 subcarriers each with $N_{Tx}=1$ and $N_{Rx}=3$ (1×3 MIMO system). We conduct experiments using 802.11n-based CSI Tool as described in Reference [41] on the receiver to acquire WiFi CSI measurements on 30 subcarriers. We have used MATLAB R2016a for signal processing throughout our experiments.

We test the proposed prototype in two cluttered scenarios, given as:

- Scenario-I (Actual driving): In this scenario, attentive activities are performed with actual driving a vehicle. Due to safety purposes, inattentive activities are performed by parking the vehicle on a side of the road.
- Scenario-II (Vehicle standing in a garage): In this scenario, all prescribed activities are performed inside a vehicle while standing in a garage of size 18×20 feet.

**Figure 7:**
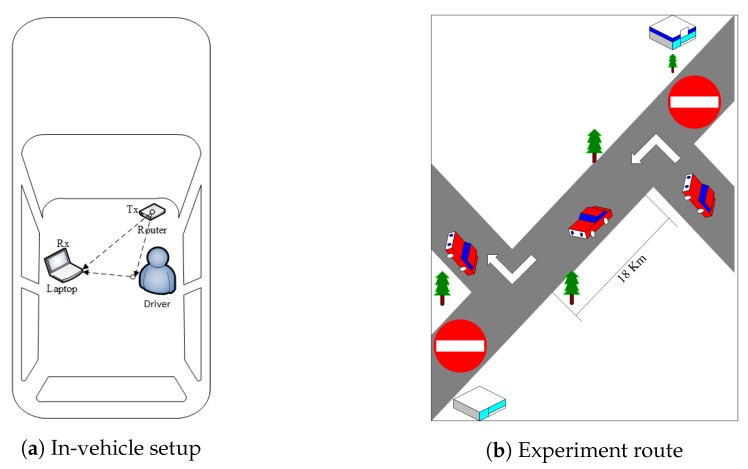
Experimentation settings.

For in-vehicle scenarios, we set up testbed in a locally manufactured vehicle that was not equipped with pre-installed WiFi devices. Due to the unavailability of a WiFi access point in the test vehicle, we configured a TP-Link (TL-WR742N) router as AP, placed on the dashboard in front of the driver. The receiver is placed at co-pilot’s seat to collect CSI data of the WiFi signal, as demonstrated in Figure 7a. For scenario-I, the vehicle is derived on a road of about 18 km long (with left and right turns on both ends) inside the university campus with an average speed of 20 km/h, as shown in Figure 7b.

**Figure 8:**
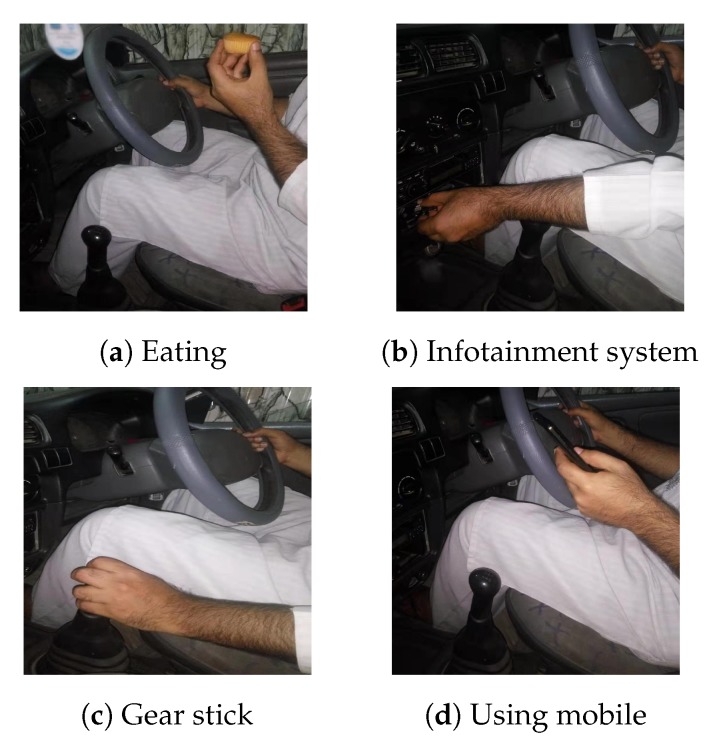
Activities performed.

In our experiments both attentive and inattentive activities are considered to get detailed classification results. For attentive class, we considered driving maneuvers and other primary activities that are necessary for driving, while distraction and fatigue are regarded as an inattentive class, as shown in Table 1. In each experiment scenario, 15 possible driver’s activities are performed (4-driving maneuvers, 2-primary activities, 5-distraction activities, and 4-fatigue activities). Some pre-defined activities are shown in Figure 8. Five volunteers (3-males and 2-females university students) were deployed to perform the activities, who do not know very much about activity recognition. Each activity is performed within a window of 5 seconds and repeated 20 times by each volunteer. During the performance of pre-defined activities, no other activity is performed to avoid interference. In total, the data set comprising of 1500 samples (15-activities × 5-volunteers × 20-times repeated) for each experiment scenario; of which 50% are used for training and 50% for testing. In our experiments, the training data do not contain the samples from testing data, and we keep the testing samples out for cross-validation.

### 5.2. Performance Evaluation

In this section, we will evaluate the performance of proposed framework. For simplicity, we use abbreviated terms for the suggested feature extraction method, i.e., the combination of Gabor and GLCM features from discriminant component selection, as GC. We used the Stacked Sparse Auto-Encoder (S-SAE) model in all experiments unless it is mentioned. GC feature extraction scheme with the S-SAE algorithm is abbreviated as GC-S.

**Figure 9:**
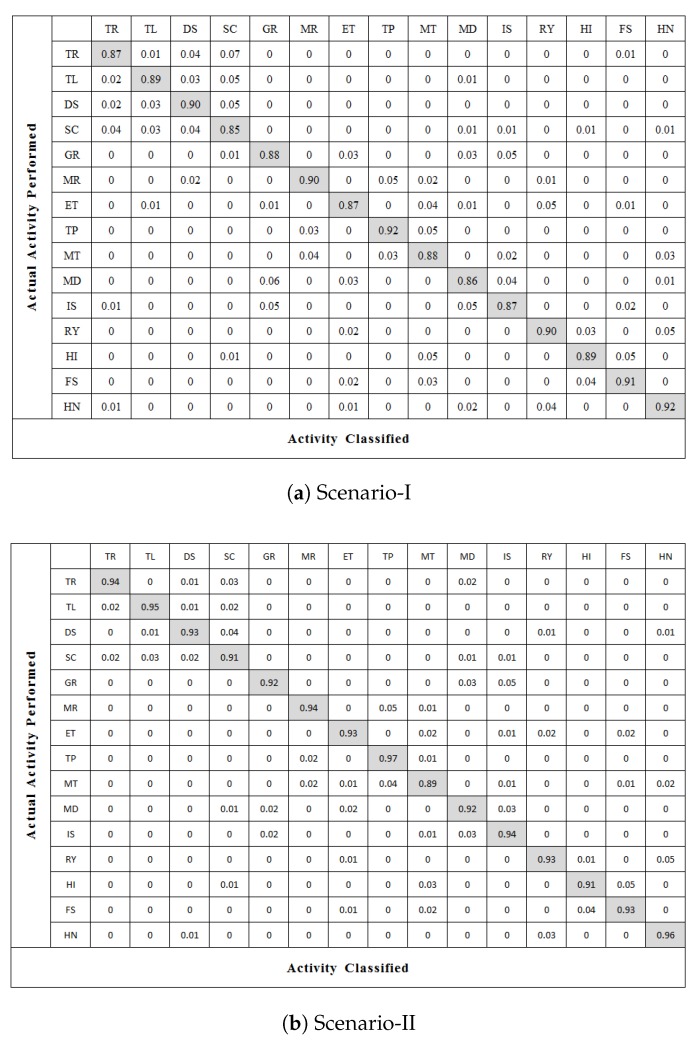
Confusion matrix of activity recognition using GC-S algorithm.

We particularly select recognition accuracy and confusion matrix to show the obtained results. The occurrence of actual activity performed is represented by the column of the confusion matrix, whereas the occurrence of activity classified is represented by the row. From the confusion matrix in Figure 9, it is clear that the proposed GC-S algorithm can recognize fifteen activities very accurately with an average recognition rate of 88.7% and 93.1% for scenarios I and II respectively.

To validate the efficacy and reliability of the proposed prototype, we investigate the obtained results using state-of-the-art evaluation metrics including precision, recall, and $F_{1}$-score. We abbreviate false positive, true positive and false negative as FP, TP and FN respectively, then these evaluation metrics are mathematically defined as [56]:

(33) $$Precision=\frac{TP}{TP+FP}$$

(34) $$Recall=\frac{TP}{TP+FN}$$

(35) $$F_{1}=2\times \frac{Precision\times Recall}{Precision+Recall}$$

**Figure 10:**
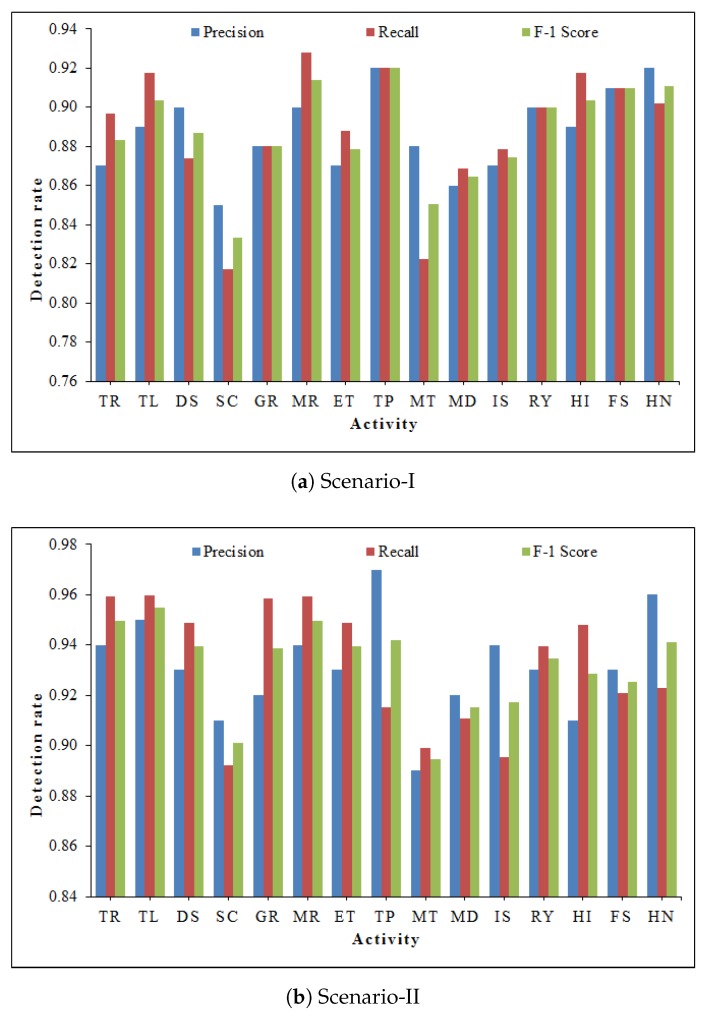
Precision, recall and F-1 score for each activity using GC-S method.

The results related to precision, recall, and $F_{1}$-score using proposed GC-S method are illustrated in Figure 10. It can be seen that both scenarios have acceptable performance using GC-S method. The average minimum and maximum values are summarized in Table 2.

**Figure 11:**
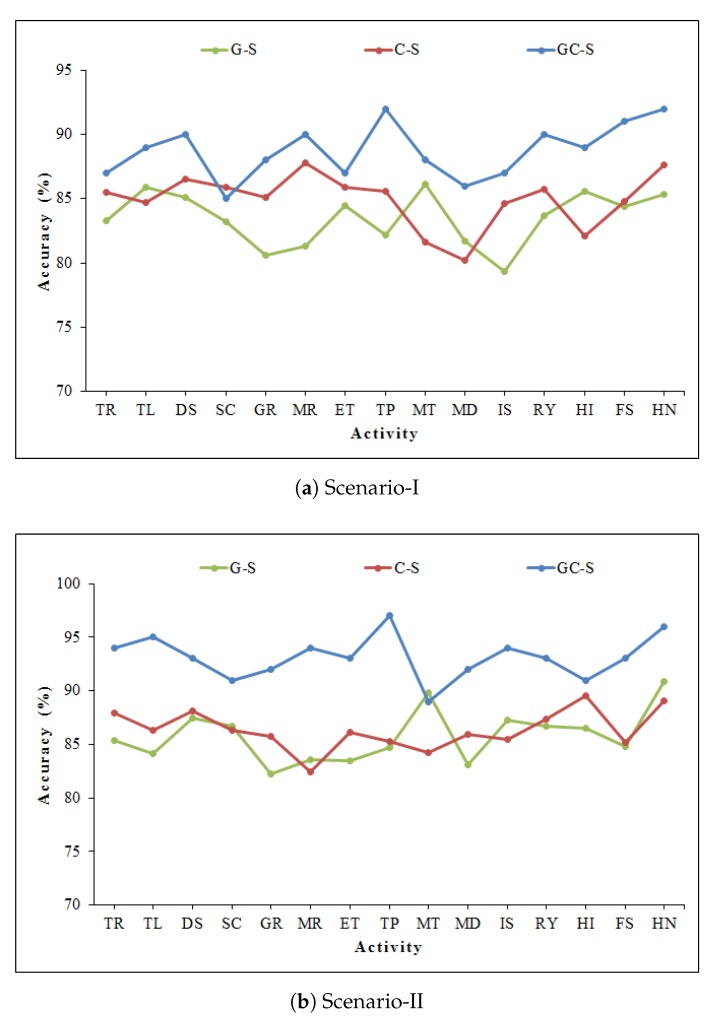
Comparison of accuracy for each activity with stand-alone features.

We have compared the accuracy of GC-S algorithm with stand-alone feature extraction methods, i.e., Gabor and GLCM features are extracted separately from CSI-transformed image (after discriminant components selection). Gabor method with S-SAE model is abbreviated as G-S, while the GLCM method with S-SAE is abbreviated as C-S. As can be seen in Table 3, the proposed GC-S method has the best performance in comparison to stand-alone Gabor (G-S) and GLCM (C-S). The detailed comparison for each activity recognition is described in Figure 11.

**Figure 12:**
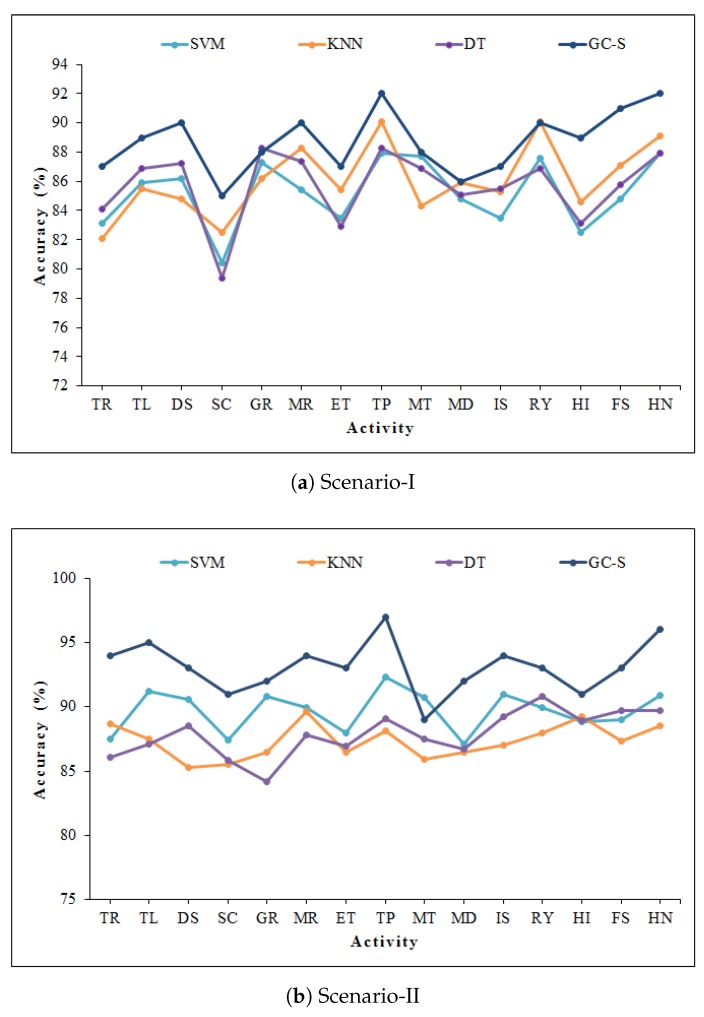
Classification performance of GC-based features with conventional classifiers.

For the robustness evaluation of the proposed scheme, we investigate the recognition accuracy of suggested GC feature extraction algorithm with widely used state-of-the-art classifiers including Support Vector Machine (SVM), K Nearest Neighbors (KNN), and Decision Tree (DT). For the implementation of SVM and KNN classifier, we follow the procedure as described in Reference [25]. For KNN, the presented system accomplishes the most accurate recognition performance at K = 3 nearest neighbors. For DT classifier, we adopt C4.5 algorithm as described in Reference [57]. From the results highlighted in Figure 12, it can be concluded that GC features have reasonable performance using conventional classifiers, but comparatively better results are obtained when using with S-SAE. This observation validates the significance of the S-SAE algorithm employed in classification scheme. The overall results are summarized in Table 4.

**Figure 13:**
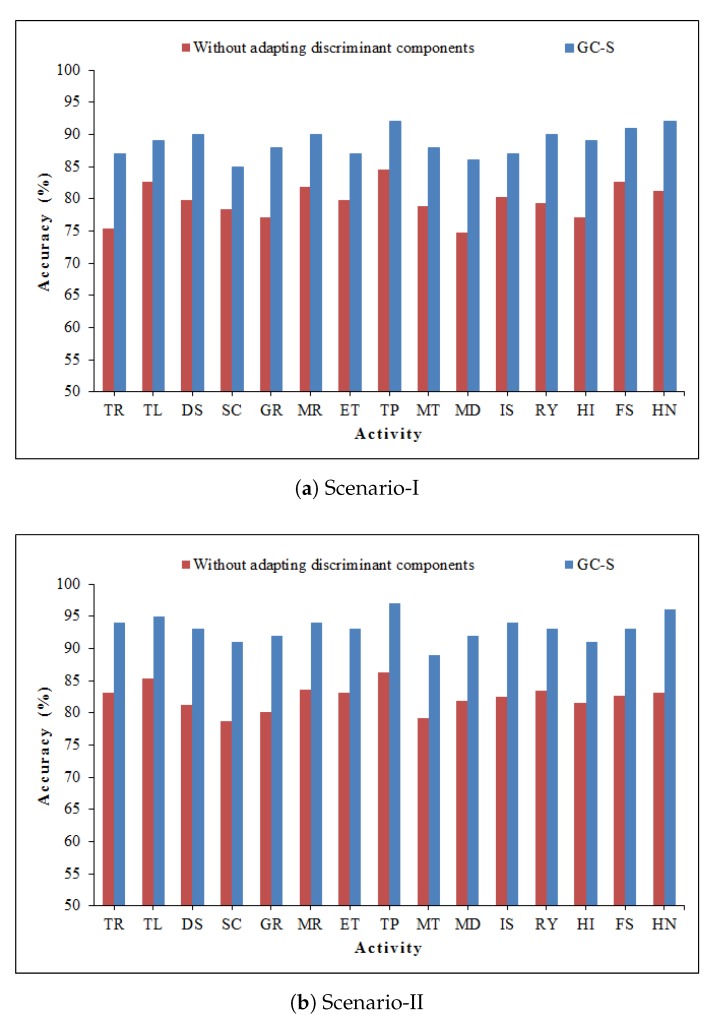
Comparison of accuracy without adapting discriminant component selection method.

To ensure the importance of discriminant components selection, we checked the recognition accuracy using all the information of radio-images. For the purpose, Gabor and GLCM features are extracted from the whole image (without adapting the discriminant component selection method). As expected, a significant drop in recognition accuracy is observed. Figure 13 reveals the fact that the recognition accuracy drops to 79.6% and 82.4% for scenario-I and II, respectively. One can notice that discriminant components are playing a vital role in recognition accuracy.

**Figure 14:**
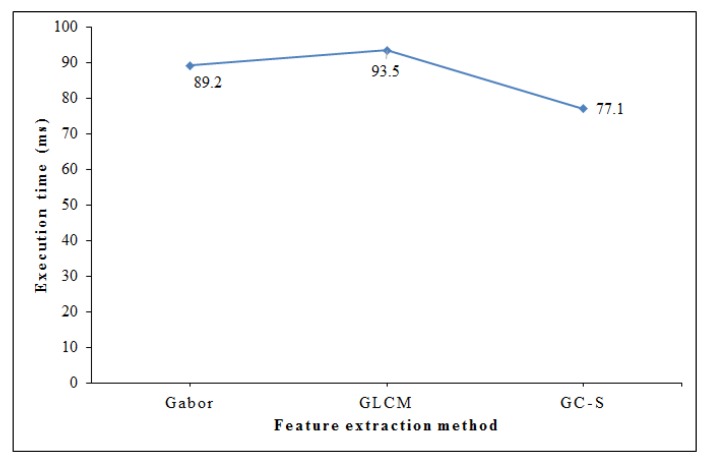
Comparison of execution time.

We have calculated the execution time to evaluate the computational efficiency of proposed system, as shown in Figure 14. The stand-alone Gabor and GLCM features are extracted without adapting discriminant components selection. In general, GC-S has relatively less execution time of 77.1 ms as compared to Gabor with execution time 89.2ms and GLCM method with 93.5 ms. Thus, it proves the computational efficiency of proposed GC-S algorithm with less execution time. Table 5 demonstrates the execution time for each processing module of suggested framework. The major time is consumed in activity profile extraction and discriminant components selection that is still acceptable as the most significant part of presented model. Moreover, it is evident from Table 3 that the recognition accuracy of GC-S is comparatively better as compared to conventional Gabor and GLCM features. Hence, the proposed GC-S scheme is low-cost solution with high recognition performance.

**Figure 15:**
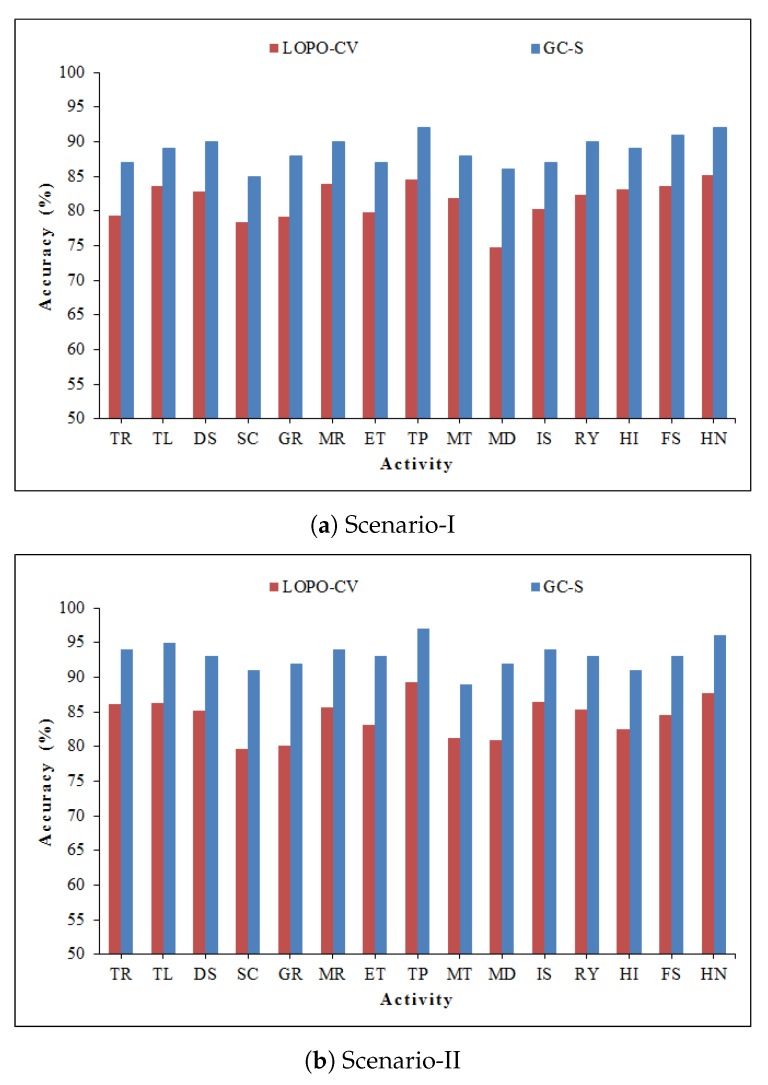
Accuracy test using LOPO-CV scheme.

The training data has a vital impact on the results in terms of variation in classification accuracy. To verify this hypothesis, we performed a user independence test. We specifically adopt the Leave-One-Person-Out Cross-Validation (LOPO-CV) scheme [58]. In this generalization technique, the test-user data is not included in training data. In particular, all data is considered to be the training data set, except a specific personś data that is selected as the test-user. This process is repeated for each entity individually until all users are treated as test-user. The results related to the LOPO-CV scheme are enclosed in Figure 15. The presented algorithm has an acceptable performance with an average recognition accuracy of 81.5% and 84.3% for scenarios I and II, respectively. One can conclude that the proposed method has a generalization capability for new users.

To evaluate the performance of presented radio-image-based system, we compare the recognition accuracy of proposed GC feature extraction technique with the conventional time-domain and frequency-domain feature extraction method [59]. We particularly select three widely used time-domain features including mean, variance and peak-to-peak value, and two commonly used frequency-domain features including entropy and energy. From Table 6, it is clear that the proposed GC feature extraction technique outperforms as compared to conventional methods.

**Figure 16:**
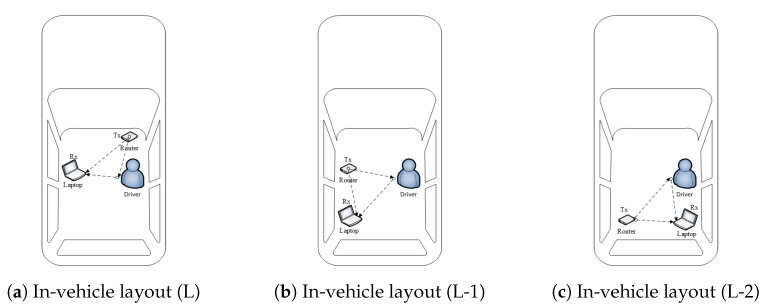
Varying layout.

The proposed algorithm is tested with three different placements of transmitter and receiver, i.e., layout L (actual layout), L-1, and L-2, as shown in Figure 16. From the results described in Table 7, it is obvious that the presented mechanism is independent of in-vehicle layout, with acceptable recognition performance at all placements of transmitter and receiver.

## 6. Discussion

In this section, we will discuss the potential results and limitations. Although WiFi-based models can approach a reasonable recognition accuracy, some limitations still exist. Firstly, the CSI of WiFi signals is very sensitive to moving objects, the motion of other vehicles in the testing area may result in degradation of system performance. Furthermore, we considered only the driver’s activities in a vehicle; however in the actual scenario, there may be multiple passengers in the vehicle that can affect the recognition accuracy. Thus, additional signal processing and advanced filtering techniques may be applied to combat these issues that will be considered in future research. Moreover, the driver may perform two activities simultaneously that is not considered in this research study. There are also several important factors needed to be considered in future studies, such as personalized driving habits and the impact of driver orientation on recognition performance. These limitations can be overthrown with a wide range of samples in training data.

From the experimental evaluation, it is clear that all activities have been classified with very good recognition accuracy using the proposed GC-S algorithm. However, some limiting factors may affect the recognition accuracy of the system. One important fact is that some activities have close resemblance with each other, e.g. the head-nodding activity has resemblance to repeated yawning, and head itching has some resemblance with face scratching. Furthermore, each driver performs activities differently, e.g. in repeating yawning some drivers may open the only mouth but in other cases he/she may use the hand as well during yawning. Despite all these limitations, the WiFi-based driver’s activity recognition system is more accurate and easy to deploy as compared to conventional methods.

In general, the proposed GC feature extraction method is independent of a classifier. However, we noticed a remarkable performance improvement using the GC feature extraction framework with the S-SAE model. We can conclude that all classifiers have good recognition accuracy using the presented computationally efficient feature extraction algorithm. We have also observed that the average recognition accuracy of the GC-S method is much better as compared to G-S or C-S that makes the GC-S method preferable. Although the system has enough recognition performance using Gabor or GLCM features alone. However, to ensure the reliable performance of the system and maximize the recognition accuracy, we are using both Gabor and GLCM features.

For instance, the impact of the change of testing vehicles has not been examined that we will consider in future work. This can be accomplished either by traditional extensive training of the classifier, or by adopting more advanced mechanisms [60]. The presented GC-S scheme is derived from the basic feature extraction and classification research that is a general solution to solve any device-free localization and activity recognition problem. In this paper, we implemented this method for driver monitoring. The promising results suggest that this approach is an effective candidate for advanced automotive applications.

It should be mentioned that the presented research work is concerned with the performance of WiFi-based in-vehicle activity recognition, but it is not obvious that WiFi devices could get deployed in the future, or even whether they should be pre-installed in smart vehicles. Furthermore, we implemented this prototype on a conventional vehicle by deploying a WiFi access point inside the vehicle; however in future smart vehicles, WiFi router’s antenna may be installed outside the vehicle that may degrade the system performance. To overcome this limitation, we propose to use beam-forming techniques at the receiver and strengthen the WiFi signals reflected from the driver’s body. However, the suggested framework is robust and the overall accuracy does not change much for varying placements of WiFi router inside the vehicle.

## 7. Conclusions

In this paper, we presented a simple yet effective mechanism for WiFi-based driver’s activity monitoring with efficient computation of Gabor and GLCM features. It can be concluded that the discriminant components play a vital role in any activity recognition framework. Compared with traditional techniques, the proposed solution is computationally efficient with less execution time and has achieved a significant improvement in recognition accuracy. It is a generalized solution that can be used for other localization and activity recognition systems as well. The proposed system is easily deployable, robust and scalable in any environment. The presented framework may be a significant component for a wide range of applications including driver safety and assisted driving systems for intelligent vehicles.

The proposed model powerfully demonstrated the performance of the driver monitoring system based on obtained outcomes. However, there are still in-depth investigations required to carry out in the future with efficient feature extraction methods for more complex driving scenarios. It may be extended to multiple passengers by posing additional signal processing challenges.

## Figures and Tables

**Table 1:** Proposed activities description.

Driver State	Activity Type	Activity Label	Activity Performed
Attentive	Driving maneuvers	TR	Turning Right
		TL	Turning Left
		DS	Driving Straight
		SC	Steering Corrections
	Primary tasks	GR	Operating Gear Stick
		MR	Mirrors Checking
Inattentive	Distraction	ET	Eating
		TP	Talking with Passenger
		MT	Talking or Listening on Mobile Phone
		MD	Dialing a Mobile Phone
		IS	Operating Infotainment System
	Fatigue	RY	Repeated Yawning
		HI	Head Itching
		FS	Face Scratching
		HN	Head Nodding

**Table 2:** Overall detection rate of precision, recall and $F_{1}$-score.

Experiment	Average Rate (%)					
	Precision		Recall		$F_{1}$-Score	
	Min.	Max.	Min.	Max.	Min.	Max.
Scenario-I	86	92	82	93	83	91
Scenario-II	89	97	89	96	89	95

**Table 3:** Comparison of GC-S with stand-alone G-S and C-S method.

Experiment	Average Recognition Accuracy (%)		
	G-S	C-S	GC-S
Scenario-I	83.5	84.9	88.7
Scenario-II	85.8	86.3	93.1

**Table 4:** State-of-art classification performance.

Experiment	Average Recognition Accuracy (%)			
	SVM	KNN	DT	S-SAE
Scenario-I	85.2	86.1	85.7	88.7
Scenario-II	89.7	87.3	87.9	93.1

**Table 5:** Execution time of processing parts per activity.

Parts	CSI Pre-Processing	Activity Profile Extraction	Feature Extraction	Classification	Total
Time (ms)	12.5	25.1	18.2	21.3	77.1

**Table 6:** Comparison of GC-S technique with conventional methods.

Experiment	Features Type	Time Domain			Frequency Domain		GC-S
		Mean	Variance	Peak-to-Peak	Entropy	Enery	
Scenario-I	Accuracy (%)	80.3	79.1	76.2	79.5	77.4	88.7
Scenario-II	Accuracy (%)	87.5	80.6	78.4	87.1	86.3	93.1

**Table 7:** Performance evaluation with varying layout.

Experiment	Average Recognition Accuracy (%)		
	L-1	L-2	L
Scenario-I	88.2	86.5	88.7
Scenario-II	90.8	89.6	93.1
